# Treating Non-fentanyl-derived Synthetic Opioid Overdose with a Traditional Opioid Reversal Agent

**DOI:** 10.7759/cureus.7241

**Published:** 2020-03-11

**Authors:** Valerie S Acuna, Ethan M Abels, Amir Alhelwani, Saeed K Alzghari

**Affiliations:** 1 Pharmacy, University of North Texas Health Science Center, Fort Worth, USA; 2 Pharmacy, Texas Tech University Health Sciences Center, Dallas, USA; 3 Pharmacy, Baylor Scott & White Medical Center, Waxahachie, USA; 4 Pharmacotherapy, University of North Texas Health Science Center, Fort Worth, USA; 5 Pharmacy Practice, Texas Tech University Health Sciences Center, Dallas, USA

**Keywords:** u47700, butyrfentanyl, opioid abuse, opioid crisis, nso, naloxone, opioid reversal

## Abstract

As the opioid crisis in the United States continues to grow, non-fentanyl-derived synthetic opioids (NSOs) are growing in both availability and popularity. NSO use comes with considerable risk including a high potential for both abuse and overdose. In this editorial, we review the consequences of overdose with the NSOs U-47700 and butyrfentanyl (BF) and the potential for the use of naloxone as a treatment for such instances. Naloxone administration was found to be successful in reversing opioid effects and re-establishing independent breathing in a patient taking U-47700 or BF. With a high rate of success in treating opioid overdose and a low chance of negatively affecting healthy non-dependent persons, naloxone is an ideal medication in these situations. We recommend the use of naloxone in cases of NSO opioid overdose and advocate for the increased availability of naloxone products to improve overdose outcomes nationwide.

## Editorial

In the United States, the current opioid crisis is dire. More than 33,000 people died due to opioid overdose in 2015, and approximately half of those cases were from prescription opioids [1]. With this growing epidemic, a rise in synthetic opioid use is taking place due to their easy accessibility compared to prescription opioids. Non-fentanyl-derived synthetic opioids (NSOs), with their high toxicity and dangerous adverse effects, are replacing heroin, bypassing drug control laws, and are illicitly manufactured and sold online, resulting in numerous abuse and overdose cases. NSOs belong to a broad family of analgesics and anesthetics acting at the mu, delta, and kappa opioid receptors [2]. The psychological and physiological side effects can vary depending on the specific opioid being used and the type of receptor that is stimulated or blocked. Currently, NSOs are not detected by conventional toxicology tests, making the detection and identification of at-risk patients even more difficult [2]. However, the ability to reverse the effects of an NSO with an agent such as naloxone can be the difference between life and death.

Currently, initial treatment for patients presenting to a hospital emergency department with signs of an opioid overdose consists of supportive care such as ventilation and reversal of the effects caused by the opioid. Opioid overdoses are caused by drugs that are strong mu-opioid receptor (MOR) agonists. An opioid overdose is characterized by respiratory depression, central nervous system depression, and pinpoint pupils [3]. In the event of a suspected opioid overdose, naloxone should be administered and titrated appropriately. Naloxone is a competitive MOR antagonist that reverses the effects of an opioid. Naloxone has a rapid onset of action and can be administered in a variety of ways including intravenously (IV), intramuscularly (IM), and intranasally (IN). Other management options for patients that present with an opioid toxidrome may include continuous intubation and the use of additional supportive medications. In addition to treatment, immunoassays are also utilized to identify the drug or chemical that is causing the reaction. While immunoassays are effective in detecting certain substances, there are many drugs that are unable or difficult to be identified by immunoassay, such as NSOs.

U-47700 is an NSO that is recognized as a schedule I drug by the Drug Enforcement Agency (DEA). A recent review evaluated the consequences of using U-47700 [3]. Most of the fatalities due to U-47700 involved patients who typically presented to the hospital with pulmonary edema or cerebral edema. Among the 16 cases reviewed, only six of those patients survived. These patients presented to the hospital with decreased mental status and decreased respiratory rate suggestive of an opioid toxidrome. Additionally, the patients presented with hypoxia, tachycardia, and respiratory depression. Naloxone was used to treat these patients and reverse the coma and bradypnea caused by U-47700.

Another NSO recognized as a schedule I drug by the DEA is butyrfentanyl (BF), an analog of fentanyl. A recent systematic review of 10 articles covering a total of 33 cases, 20 of which postmortem, revealed a trend of significant pulmonary and cardiovascular damage among patients who used BF or a similar analog [4]. Of patients that survived, many required ICU stays, with some patients needing ventilator support due to pulmonary aspiration or intubation in the course of treatment. Altered mental status, dyspnea, and hemoptysis were also seen in the patients studied. Naloxone was commonly administered in response to these overdoses and treatment was successful for 90% of patients included in the cases reviewed.

In the case of U-47700 and BF, naloxone was often given to reverse the effects of the opioid and help re-establish independent breathing. Many of the fatalities may have been preventable with timely detection and administration of naloxone. Naloxone is a well-established medicine that can be used in life-threatening situations involving opioid overdose [1]. It is opioid-specific and actively displaces opioids from the MOR. The onset of action for naloxone is rapid for IV use, only taking about a few minutes, and relatively quick for IM and IN, taking only about 15 minutes to produce its effects [1]. Naloxone does not cause any type of dependence and has minimal side effects on a healthy non-dependent person.

As cases of opioid overdoses by NSOs continue to increase, there is an urgent need for better accessibility to naloxone. Possible solutions to improving access to naloxone include dispensing take-home naloxone to opioid users or making naloxone an over-the-counter (OTC) medication in the United States. New naloxone products such as pre-filled syringes, auto-injectors, and nasal sprays are becoming increasingly available [1]. These products have been tested for safety and efficacy and have a bioavailability of 40-50%. Additionally, advances in research continue to improve and refine these products. Traditional barriers limiting naloxone's use such as legal challenges, cost, and stigma associated with its use have been minimized. The current United States Surgeon General, Dr. Jerome Adams, has urged family and friends to keep naloxone on hand for people at risk of an opioid overdose [5]. Furthermore, naloxone can be obtained as OTC medications in most states through local pharmacies and can be billed to certain insurers to cover its costs. Lastly, naloxone should be discussed with patients during counseling for prescription opioids by physicians and pharmacists to minimize the stigma associated with the drug. 

Thousands of people around the world have suffered from the malaise of opioid overdose. In the United States, deaths caused by illicit synthetic opioids like U-47700 and BF continue to increase. It is proven that faster administration of naloxone could prevent many of these deaths. Raising awareness regarding naloxone's accessibility among patients and their families may help prevent deaths caused by opioid overdoses.
